# Targeting Diabetic Atherosclerosis: The Role of GLP-1 Receptor Agonists, SGLT2 Inhibitors, and Nonsteroidal Mineralocorticoid Receptor Antagonists in Vascular Protection and Disease Modulation

**DOI:** 10.3390/biomedicines13030728

**Published:** 2025-03-17

**Authors:** Merita Rroji, Nereida Spahia, Andreja Figurek, Goce Spasovski

**Affiliations:** 1 Department of Nephrology, University of Medicine Tirana, 1001 Tirana, Albania; 2 Department of Nephrology, University Hospital Center Mother Tereza, 1001 Tirana, Albania; edaspahia01@gmail.com; 3 Institute of Anatomy, University of Zurich, 8057 Zurich, Switzerland; andrejafigurek@yahoo.com; 4 Department of Nephrology, University Sts. Cyril and Methodius, 1000 Skopje, North Macedonia; spasovski.goce@gmail.com

**Keywords:** diabetic kidney disease, atherosclerosis, endothelial dysfunction, oxidative stress, inflammation

## Abstract

Atherosclerosis is a closely related complication of diabetes mellitus (DM), driven by endothelial dysfunction, inflammation, and oxidative stress. The progression of atherosclerosis is accelerated by hyperglycemia, insulin resistance, and hyperlipidemia. Novel antidiabetic agents, SGLT2 inhibitors, and GLP-1 agonists improve glycemic control and offer cardiovascular protection, reducing the risk of major adverse cardiovascular events (MACEs) and heart failure hospitalization. These agents, along with nonsteroidal mineralocorticoid receptor antagonists (nsMRAs), promise to mitigate metabolic disorders and their impact on endothelial function, oxidative stress, and inflammation. This review explores the potential molecular mechanisms through which these drugs may prevent the development of atherosclerosis and cardiovascular disease (CVD), supported by a summary of preclinical and clinical evidence.

## 1. Introduction

Diabetic kidney disease (DKD) is a significant public health challenge, representing the leading cause of end-stage kidney disease (ESKD) globally and accounting for nearly half of all ESKD cases in industrialized nations [1]. In fact, the rising prevalence of diabetes mellitus (DM), particularly type 2 diabetes mellitus (T2D), underscores the substantial burden of DKD as a primary driver of renal failure and its associated complications [2]. The progressive nature of DKD, characterized by persistent albuminuria, declining glomerular filtration rate (GFR), and consequent renal fibrosis, contributes to the predominant representation of diabetic patients among those with ESKD [3].

In the majority of these diabetic patients, the disease trajectory remains difficult to amend, driven by persistent metabolic dysregulation, inflammation, oxidative stress, and fibrotic remodeling of renal tissues [4]. Indeed, the inability to reverse established renal damage reflects the progressive and cumulative nature of DKD pathology [5]. Fibrotic changes, central to the advanced DKD, result in an irreversible scarring of the renal parenchyma and a consequent loss of functional nephrons [3]. Simultaneously, atherosclerosis emerges as a leading cause of mortality in individuals with diabetes [6], reflecting a complex interplay of metabolic disturbances, chronic inflammation, and vascular dysfunction, all exacerbated by persistent hyperglycemia and insulin resistance that compromise vascular integrity [7].

Nowadays, current treatments for DKD primarily target risk factors such as hyperglycemia, hypertension, and dyslipidemia. However, these interventions fail to address the underlying molecular mechanisms driving DKD and its associated atherosclerotic complications [8]. This limitation underscores the pressing need for innovative therapeutic approaches for early intervention to prevent irreversible kidney damage and strategies that target fundamental pathophysiological processes underlying atherosclerosis in DKD This review discusses the role of GLP-1 receptor agonists (GLP-1 RAs), sodium–glucose cotransporter 2 (SGLT2) inhibitors, and nonsteroidal mineralocorticoid receptor antagonists (nsMRAs) in the generation and progression of atherosclerosis through their effects on endothelial function, vascular inflammation, lipid metabolism, oxidative stress, and plaque stability. In addition, it evaluates their role in reducing cardiovascular risk, including the prevention of major adverse cardiovascular events (MACEs), supported by findings from both preclinical models and clinical trials. By integrating mechanistic insights with therapeutic outcomes, this review comprehensively explains how these emerging antidiabetic agents contribute to vascular protection and atherosclerotic disease modification.

## 2. Pathophysiological Mechanisms Underlying Atherosclerosis in DKD

Atherosclerotic cardiovascular disease (ASCVD) refers to a spectrum of disorders, including acute coronary syndrome (ACS), stable angina, coronary or other forms of revascularization, transient ischemic attack, ischemic stroke, and peripheral vascular disease [9]. SCVD is responsible for approximately two-thirds of cardiovascular (CV) morbidity and fatalities globally [10]. It frequently coexists with DKD and contributes to cardiovascular complications that may significantly impact on patients’ lifespans. Individuals with T2DM tend to reveal a more diffuse distribution of cardiovascular complications compared to those without diabetes. Approximately one-third of people with T2DM have cardiovascular disease, and in 90% of these cases, the condition is atherosclerotic in nature [11]. Diabetic atherosclerosis is marked by plaque accumulation within the vascular system, driven by metabolic disturbances such as hyperglycemia, insulin resistance, and lipid abnormalities. The traditional initiatory event in its course is endothelial dysfunction through impairment of nitric oxide generation, an essential molecule regarding vascular integrity as an effect of chronic high blood sugar and oxidative stress [7].

The activating endothelial cells release inflammatory cytokines and adhesion molecules, facilitating monocyte adhesion and migration into the subendothelial space [7]. Once there, monocytes differentiate into macrophages that ingest oxidized low-density lipoprotein (oxLDL), forming foam cells as a feature of an early atherosclerotic lesion. These foam cells aggregate, forming fatty streaks that precede more advanced plaques.

As the disease progresses, smooth muscle cells migrate from the media to the intima of the blood vessel, where they proliferate and secrete extracellular matrix components. This leads to the formation of a fibrous cap over the fatty streak and the maturation of plaques. Hyperglycemia enhances this process by forming advanced glycation end-products and interacting with receptors, promoting oxidative stress, inflammation, and vascular calcification [12,13].

These pathological mechanisms lead to unstable plaques prone to rupture, increasing the risk of thrombosis and vascular obstruction that may result in life-threatening events such as myocardial infarction and stroke [14]. Vulnerable carotid plaques are a key factor in the development of ischemic stroke caused by large-artery atherosclerosis, even when the arterial lumen is narrowed less than 50%. Inflammatory cytokines, adhesion molecules, oxLDL, and enzymes that break down extracellular matrix components are closely linked to plaque instability. The interaction of these molecules drives inflammation, increases the likelihood of rupture, and contributes to blood clot formation. Due to the complexity and variability of these plaques, assessing their vulnerability requires a deeper understanding of immune-driven processes that contribute to thrombo-inflammation [15]. Chronic hyperglycemia and insulin resistance, even in asymptomatic patients [16], coupled with other risk factors such as obesity, hypertension, and dyslipidemia, play important roles in disease development [17]. Over the past few decades, numerous biochemical pathways that underlie diabetic macroangiopathy have been unraveled, indicating complex factors leading to vascular damage in individuals with diabetes.

### 2.1. Pathways Leading to Atherosclerosis and Cardiovascular Disease in DKD

#### 2.1.1. Insulin Resistance and Its Multifaceted Impacts

Insulin resistance (IR) has been firmly linked to ASCVD through three principal pathways: (i) the inherent molecular defect driving IR [18,19,20], (ii) the compensatory hyperinsulinemia produced by pancreatic β-cells in response to diminished insulin action [21], and (iii) the aggregation of metabolic derangements collectively termed as insulin resistance syndrome (IRS) [22,23,24,25]. On the molecular front, adequate insulin signaling relies on its receptor’s activation, which then triggers tyrosine phosphorylation of IRS-1/IRS-2 and subsequent phosphatidylinositol 3-kinase (PI3K) stimulation [26,27]. When the PI3K cascade is compromised, nitric oxide (NO) synthesis falls, predisposing to an endothelial dysfunction, a major contributor to a plaque formation [22,23]. Meanwhile, the increased circulating insulin levels seen with IR preferentially enhance mitogen-activated protein kinase (MAPK) activity, leading to the vascular smooth muscle cell growth, inflammation, and eventual atheroma development [19,28,29,30].

In addition, excess insulin also promotes de novo lipogenesis, fosters oxidative stress, and stimulates inflammatory mediators, intensifying vascular damage [31]. Obesity, particularly visceral adiposity, commonly underlies insulin resistance by creating an environment of chronic low-grade inflammation and elevated free fatty acids. An adipose tissue excess, particularly visceral fat, releases proinflammatory cytokines [leptin, adipsin, adiponectin, omentin, tumor necrosis factor-alpha (TNF-α), interleukin-6 (IL-6), monocyte chemoattractant protein-1 (MCP-1), plasminogen activator inhibitor-1 (PAI-1), resistin, visfatin, and retinol-binding protein 4 (RBP4)] and reduces the levels of protective adiponectin that impairs insulin signaling in the muscle, liver, and other tissues [32]. This systemic inflammatory state, coupled with the increased flux of free fatty acids, disrupts glucose uptake and worsens metabolic control, perpetuating both obesity and insulin resistance [33,34].

Moreover, obesity confers a heightened atherosclerotic risk through multiple pathways, independent of insulin resistance. Excess visceral adipose tissue disrupts adipokine balance, lowering protective adiponectin while elevating proinflammatory mediators (e.g., resistin, leptin) that enhance endothelial activation, foam cell formation, and plaque development. Impaired autophagy in obese states facilitates an unchecked lipid accumulation in macrophages, increasing lesion size. The heightened oxidative stress that is partly driven by NADPH oxidase upregulation further depletes nitric oxide and accelerates the inflammation. Dysbiosis of the gut microbiome can exacerbate systemic inflammation by promoting pro-atherogenic immune signaling. Obesity also contributes to marked endothelial dysfunction, with reduced eNOS expression and increased ET-1 levels impairing vascular tone and favoring a prothrombotic environment. Finally, NLRP3 inflammasome activation in adipose tissue macrophages amplifies the release of IL-1β and other inflammatory cytokines, linking the obesity-driven inflammation directly to the plaque progression [35]. These changes contribute to a chronic state of low-grade inflammation, impairing endothelial function and increasing oxidative stress [35,36].

In addition, nonalcoholic fatty liver disease (NAFLD), recently renamed as Metabolic Dysfunction-Associated Steatotic Liver Disease (MASLD), frequently coexists with insulin resistance (IR) and correlates with increased cardiovascular risk. MASLD and atherosclerosis frequently co-occur because they both result from a familiar metabolic milieu that involves obesity, insulin resistance, and dyslipidemia. When subcutaneous adipose tissue cannot adequately expand, excess lipids shift to the visceral depots and the liver, heightening IR. This insulin-resistant state fosters atherogenic dyslipidemia, notably elevated triglycerides, and small, dense LDL particles, while triggering a proinflammatory environment that damages the endothelium producing plaque development. Proinflammatory cytokines released by visceral fat, together with the oxidative stress and alterations in the gut microbiota, further amplify vascular injury. Consequently, MASLD, which is often present in individuals with obesity and type 2 diabetes, serves as a hepatic indicator of metabolic derangements that accelerate atherosclerotic processes, underscoring the strong cardiovascular ties between fatty liver disease and vascular pathology. Although MASLD typically arises alongside metabolic syndrome (MetS), it is suggested that it confers additional atherogenic effects—evidenced by more vulnerable plaques and diminished endothelial progenitor cells—indicating MASLD’s distinct contribution to cardiovascular disease beyond the MetS alone [33,37,38]. All the mentioned processes collectively enhance the vulnerability of the vascular endothelium to atherogenic insults [26].

Consequently, measures that improve insulin sensitivity or reduce compensatory hyperinsulinemia are essential for mitigating the broad spectrum of IR-related cardiovascular complications.

#### 2.1.2. Chronic Hyperglycemia and Biochemical Pathway Activation

Hyperglycemia appears to cause more severe damage to endothelial cells than other cell types. Under normal physiological conditions, glucose enters endothelial cells primarily via the GLUT-1 transporter, facilitating ATP production predominantly through glycolysis. The activity of GLUT-1 is tightly regulated by extracellular glucose concentrations [39,40]. However, in individuals with diabetes, GLUT-1 activity is elevated, leading to an increased glycolytic rate, raising intracellular glucose levels, and generating advanced glycation end products (AGEs). The accumulation of AGEs has been linked to an increased endothelial cell permeability, inhibition of endothelial nitric oxide synthase (eNOS) activity, and significant damage to cellular DNA and proteins [40].

Additionally, hyperglycemia triggers endothelial cell proliferation by activating several proinflammatory and growth factor pathways. These include hepatocyte growth factor (HGF), vascular endothelial growth factor (VEGF) receptor-ligand family, Erb-B2 receptor tyrosine kinase 4 (ErbB4), insulin-like growth factor-1 (IGF-1), bone morphogenetic proteins (BMPs), nuclear factor of activated T-cells (NFAT), signal transducer and activator of transcription 3 (STAT3), nuclear factor-kappa B (NF-κB), p70S6K, and hypoxia-inducible factor-1α (HIF-1α) pathways [41]. Hyperglycemia further enhances the expression of proinflammatory molecules such as monocyte chemoattractant protein-1 (MCP-1) and the NLRP3 inflammasome while promoting mitochondrial oxidative stress and apoptosis [42].

Conversely, hyperglycemia suppresses protective, anti-inflammatory mechanisms by reducing the expression of JunD, as well as the critical scavengers of reactive oxygen species (ROS) like superoxide dismutase 1 (SOD1) and aldehyde dehydrogenase 2 (ALDH2) [43]. These changes result in eNOS uncoupling, leading to an overproduction of superoxide anions (O_2_^•−^). The impairment of NO production and eNOS uncoupling exacerbates oxidative stress and endothelial dysfunction, establishing a self-perpetuating detrimental cycle [44].

In addition to eNOS uncoupling, AGE-induced signaling pathways trigger inflammatory cascades that upregulate growth factors, leading to an endothelial cell apoptosis, increased permeability, and vascular leakage. This process facilitates cytokine responses and leukocyte adhesion, accelerating the formation and progression of atherosclerotic plaques. Moreover, the ROS facilitates LDL oxidation, a precursor to foam cell formation and plaque development [45,46].

#### 2.1.3. Dyslipidemia: The Role of Lipid Abnormalities

The development of atherosclerosis in diabetes is closely linked to the presence of small dense LDL (sdLDL), recognized as a major contributor to cardiovascular risk. While native LDL particles do not inherently promote atherogenesis, they become harmful following modifications such as desialylation, oxidation, and structural alterations [47]. These changes enhance their retention in the subendothelial space, where modified LDL binds to proteoglycans. This interaction prolongs their presence and increases uptake through non-specific phagocytosis, resulting in cholesterol build-up within cells, the formation of foam cells, and the progression of plaques. The proinflammatory and oxidative conditions associated with diabetes further accelerate these processes. Additionally, elevated levels of remnant lipoproteins and lipoprotein(a) in diabetes intensify vascular inflammation and promote coagulation, amplifying the risk of atherosclerotic complications [7].

#### 2.1.4. Chronic Inflammation and Immune Dysregulation

Inflammation is a central driver of atherosclerosis in diabetes, linking aberrant glucose metabolism with immune-mediated vascular injury [48,49]. In hyperglycemic environments, harmful signals known as damage-associated molecular patterns (DAMPs) and pathogen-associated molecular patterns (PAMPs) incite a profound inflammatory response [50,51]. These molecular triggers are recognized by membrane-bound and intracellular receptors, particularly Toll-like receptors (TLRs) and NOD-like receptors (NLRs) on macrophages, neutrophils, and dendritic cells [52]. Activation of the key NLRP3 inflammasome leads to a conversion of pro–interleukin-1β (IL-1β) and pro–interleukin-18 (IL-18) into their active forms, intensifying inflammatory cascades and provoking pyroptotic cell death in the arterial wall [53,54].

Nevertheless, adipose tissue, now recognized as an endocrine organ, secretes multiple adipokines (e.g., leptin, adiponectin, visfatin) and inflammatory mediators (TNF-α, IL-6, MCP-1) that foster insulin resistance and endothelial dysfunction [55]. In obesity, enlarged adipocytes undergo necrosis, prompting macrophage infiltration and the formation of “crown-like” structures, which can constitute up to 40% of adipose tissue cells in severely obese individuals [56]. Moreover, these infiltrating macrophages release additional chemokines, reinforcing a proinflammatory milieu that exacerbates vascular damage [57].

Concurrently, metabolic derangements in diabetes, such as elevated free fatty acids and increased oxidative stress, contribute to endothelial dysfunction. AGEs further amplify this process by binding to the receptor for advanced glycation end products (RAGE), thus increasing ROS production and perpetuating the chronic inflammation [58,59]. Within atherosclerotic lesions, macrophages transform into foam cells through the uptake of modified LDL, stimulating the additional secretion of proinflammatory cytokines [60,61]. Excess visceral adipose tissue aggravates this situation by releasing adipokines (e.g., TNF-α, IL-6) that worsen insulin resistance and foster vascular damage [62].

Along with the progressive inflammation, there is a formation of neutrophil extracellular traps (NETs), which drives both atherogenesis and thrombosis [54]. Meanwhile, IL-6 stimulates the liver to produce acute-phase reactants such as C-reactive protein (CRP) and fibrinogen, further fueling a prothrombotic state [53]. This ongoing inflammatory cycle is reinforced by the continuous generation of DAMPs from necrotic or apoptotic cells and by uncontrolled blood glucose levels [42,63]. Ultimately, the convergence of these factors—NLRP3 activation, proinflammatory adipokines, AGE–RAGE interactions, and endothelial dysfunction—explains why individuals with diabetes face an enhanced atherosclerotic risk. Novel therapeutic strategies, including targeting the NLRP3–IL-1β axis, aim to curb this inflammatory burden and potentially slow the progression of diabetes-related vascular disease [7,64,65].

Growing evidence suggests that gut microbiota imbalances (“dysbiosis”) contribute to both diabetic kidney disease (DKD) and atherosclerosis by driving low-grade inflammation and metabolic dysfunction [66,67,68]. In these conditions, a reduced abundance of anti-inflammatory bacteria (e.g., Roseburia, Faecalibacterium, Lachnospiraceae) coincides with an overgrowth of proinflammatory strains, exacerbating vascular and renal damage. Microbial metabolites—particularly short-chain fatty acids (SCFAs) and trimethylamine N-oxide (TMAO)—further modulate the host metabolism and immune pathways, potentially influencing the progression of DKD and atherosclerosis. Although animal studies have shown encouraging effects of targeting specific microbes or administering probiotics, consistent human data are lacking [69,70].

#### 2.1.5. Endothelial Dysfunction: The Epicenter of Vascular Damage

The idea that endothelial damage plays a role in the development of vascular diseases, which contribute to increased morbidity and mortality, has been recognized for many years. However, detailed exploration of the mechanisms driving this process is relatively recent and continues to evolve. Endothelial cells are essential for numerous bodily physiological functions and exhibit vascular-specific heterogeneity [71]. Beyond its role as a physical barrier regulating cellular permeability, the endothelium also performs critical autocrine, paracrine, and endocrine functions [72]. These include controlling blood flow and pressure, regulating hemostasis and coagulation, mediating immune and inflammatory responses, and facilitating vasculogenic and angiogenesis.

Endothelial dysfunction is a pivotal event in atherosclerosis development [72]. In diabetes, endothelial cells exhibit an increased expression of adhesion molecules such as vascular cell adhesion molecule-1 (VCAM-1), facilitating monocyte adhesion and migration into the intima [73]. Hyperglycemia and oxidative stress impair eNOS activity, reducing NO bioavailability and predisposing the vasculature to vasoconstriction and thrombosis. Additionally, endothelial cells exposed to high glucose undergo senescence, compromising vascular repair mechanisms [74].

#### 2.1.6. Polyol and Hexosamine Pathways in Metabolic Stress

The polyol pathway, as discussed earlier, metabolizes excess glucose into sorbitol and fructose through aldose reductase, depleting NADPH and reducing the antioxidant capacity of glutathione. First, glucose is reduced to sorbitol, which subsequently converts to fructose by sorbitol dehydrogenase, using NAD+ as a cofactor [75]. Within the diabetic setting, this pathway accelerates oxidative stress through multiple mechanisms. NADPH, essential for replenishing reduced glutathione (GSH), becomes depleted when sorbitol is formed, while NADH generated during fructose synthesis drives superoxide production [76,77]. Furthermore, aldose reductase (AR) in this pathway contributes to diabetic complications; its overexpression in ApoE knockout mice with diabetes hastens atherosclerosis, whereas pharmacological inhibition of AR alleviates disease progression [78]. AR amplifies inflammatory responses via NF-κB and activator protein-1 signaling [79,80]. Beyond polyol pathway activation, hyperglycemia can shunt glucose into the hexosamine pathway, impairing eNOS and downregulating the protective protein A20, thereby exacerbating vascular dysfunction [77,81,82].

#### 2.1.7. Uremic Toxins

The progressive decline in renal function in DKD is marked by pervasive endothelial dysfunction and a heightened enhanced risk of atherosclerosis [83,84,85]. This stems mainly from the accumulation of uremic toxins, which create a proinflammatory and prothrombotic environment, impair antioxidant defenses, and weaken endothelial repair mechanisms [86]. The endothelium responds by overexpressing adhesion molecules (ICAM-1, VCAM-1, and selectins), facilitating leukocyte infiltration and accelerating vascular injury [84,85,86].

Heightened oxidative stress, driven by excess ROS and reduced antioxidant capacity, amplifies the tissue damage and endothelial cell apoptosis [87,88,89]. Structural alterations such as cytoskeletal remodeling and cell–cell junction disruption further compromise endothelial barrier integrity. Endothelial microparticles (EMPs), released under these conditions, intensify inflammation, promote procoagulant activity, encourage smooth muscle cell proliferation, and contribute to vascular calcification [90,91].

Additionally, certain uremic toxins diminish nitric oxide (NO) bioavailability, weakening its antiplatelet and vasodilatory effects [92].

While renal function declines, abnormalities in mineral metabolism (CKD-MBD) accelerate atherosclerosis and increase the mortality beyond traditional risk factors [84,85,86]. Diminished α-Klotho—linked to both declining eGFR and protein-bound uremic toxins—raises fibroblast growth factor 23 (FGF23) levels, worsening vascular dysfunction and left ventricular hypertrophy [93,94,95]. Inadequate α-Klotho fails to control phosphate, leading to hyperphosphatemia and amplified vascular injury [96]. These interrelated defects in α-Klotho, FGF23, and phosphate ultimately drive atherogenesis: they accelerate smooth muscle cell migration, calcification, senescence, and leukocyte–endothelial interactions, ultimately destabilizing plaques [95,97,98,99].

#### 2.1.8. “Metabolic Memory” and Long-Term Vascular Risk

The concept of “metabolic memory” highlights the enduring effects of an early hyperglycemic exposure on chronic diabetic complications, including DKD [100]. This phenomenon, first identified in the Diabetes Control and Complications Trial (DCCT) [101] and its follow-up Epidemiology of Diabetes Interventions and Complications (EDIC) study, revealed that even after HbA1c levels were equalized between intensive and conventional treatment groups, the adverse impact of prior hyperglycemia persisted, significantly increasing the risk of complications [102].

Recent studies have shed light on molecular drivers of metabolic memory, with p21 emerging as a key regulator. Persistently high expression of p21 under hyperglycemic conditions promotes processes like tubulointerstitial fibrosis in DKD. Experimental inhibition of p21 has shown potential in mitigating these fibrotic changes, suggesting promising therapeutic implications [103].

**Figure 1:**
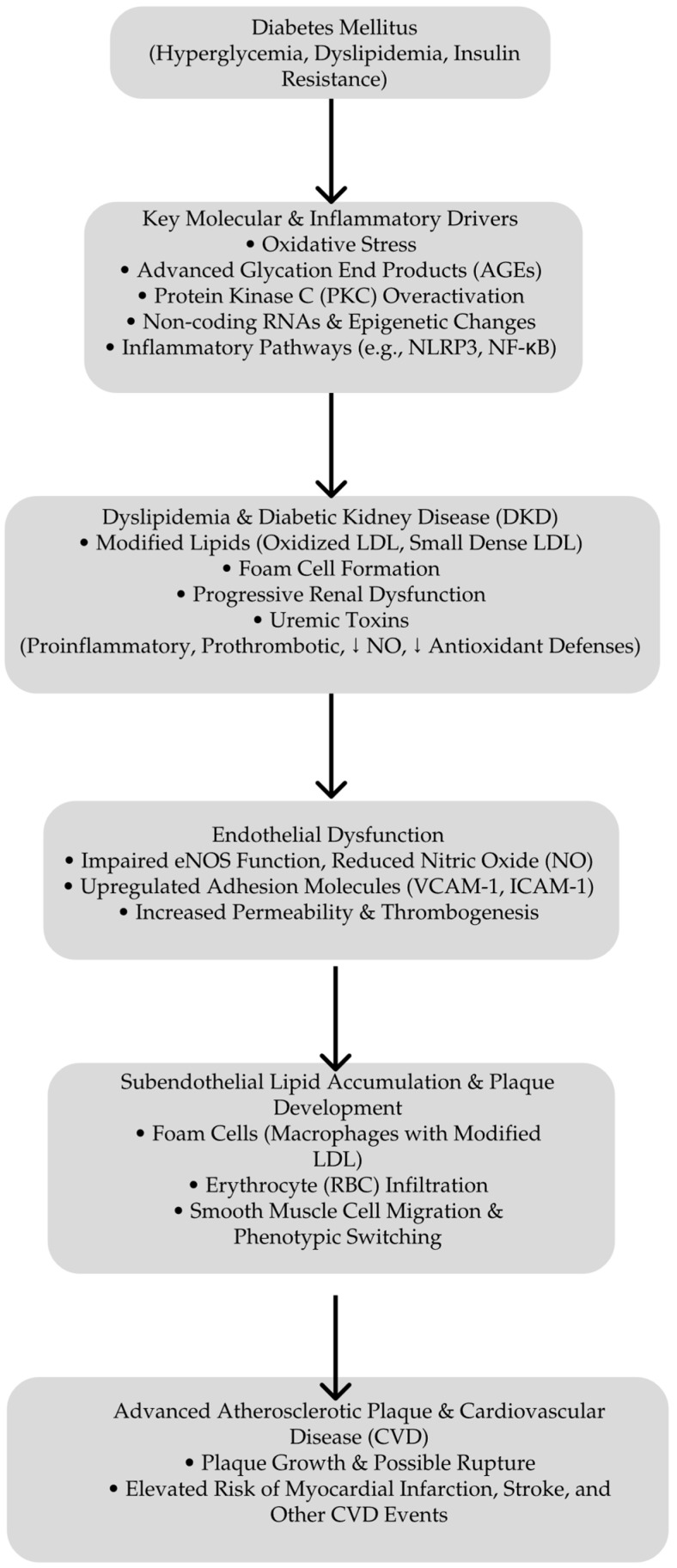
Pathways leading to atherosclerosis in DKD. Starting with hyperglycemia, dyslipidemia, and IR, diabetes triggers key molecular and inflammatory pathways—such as the oxidative stress, advanced glycation end product (AGE) formation, overactivation of protein kinase C (PKC), and non-coding RNA/epigenetic changes. These processes contribute to DKD, the generation of uremic toxins, and modified lipids. Endothelial dysfunction follows the marked impairment of NO production and increased expression of adhesion molecules facilitating subendothelial lipid accumulation, foam cell formation, and smooth muscle cell migration. This culminates in advanced atherosclerotic plaque growth and, ultimately, an elevated risk of myocardial infarction, stroke, and other cardiovascular disease events. ↓ deacrease.

Central to metabolic memory are mechanisms involving long-lived AGEs and sustained oxidative damage. These factors explain the enhanced vascular and renal risks in patients with a history of poor glycemic control, even after achieving improved metabolic regulation. This underscores the necessity of an early and intensive glycemic management to prevent the establishment of metabolic memory and reduce long-term complications [104]. Figure 1 presents the pathways that lead to atherosclerosis in DKD.

## 3. Glucagon-like Peptide-1 Receptor Agonists: A Potential Therapy for Atherosclerotic Risk in Diabetic Kidney Disease

Glucagon-like peptide-1 (GLP-1) is an intestinal hormone integral to glucose homeostasis, functioning as an insulinotropic agent by stimulating insulin secretion from pancreatic β-cells in a glucose-dependent manner while inhibiting glucagon release from α-cells. Additionally, GLP-1 slows gastric emptying and regulates appetite by enhancing satiety signals in the central nervous system [105]. The GLP-1 receptor (GLP-1R), abundantly expressed within the cardiovascular system, mediates both the metabolic and cardiovascular effects of GLP-1 [106]. Due to the rapid degradation of native GLP-1 by dipeptidyl peptidase-4 (DPP-4), resulting in a short half-life, GLP-1 receptor agonists (GLP-1RAs) have been developed to provide a more effective and sustained therapeutic option for diabetes management [107].

Extensive multinational cardiovascular outcome trials (CVOTs) have consistently demonstrated that GLP-1RAs not only enhance glycemic control but also confer significant cardiovascular benefits, including reductions in cardiovascular mortality independent of their glucose-lowering effects [108]. GLP-1RAs are available in both short-acting formulations, such as lixisenatide and exenatide, which primarily reduce postprandial glucose levels by delaying gastric emptying, and long-acting formulations, including liraglutide, dulaglutide, semaglutide, and albiglutide, which effectively enhance insulin secretion, suppress glucagon release, and lower both fasting and postprandial glucose concentrations [109].

Chronic hyperglycemia and acute glucose spikes impair endothelial function, contributing to vascular damage and the progression of CVD [110]. While many antidiabetic agents effectively lower blood glucose, not all demonstrate corresponding reductions in cardiovascular risk, underscoring the necessity for treatments that provide direct cardiovascular benefits alongside glycemic control [111]. GLP-1RAs offer such benefits by stabilizing and mitigating the progression of ASCVD through mechanisms that extend beyond glucose regulation [112]. Beyond their metabolic effects, GLP-1RAs offer profound cardiovascular protection by stabilizing and mitigating ASCVD progression. Apart from managing glucose levels, weight loss, and improving insulin resistance [111,113], these effects are also attributed to a combination of mechanisms, such as the effect on endothelial health, reduction in systemic inflammation, stimulation of nitric oxide synthesis in endothelial cells, preserved mitochondrial function, decreased oxidative stress, and improved function of the smooth vascular muscle cells [114,115].

### 3.1. Preclinical Insights into the Anti-Atherosclerotic Properties of GLP-1 Receptor Agonists in Diabetic Kidney Disease

GLP-1RAs, including exendin-4, lixisenatide, liraglutide, semaglutide, and various GLP-1 fragments, provide comprehensive protective effects against atherosclerosis across multiple stages of plaque development [116] (see Table 1). A crucial aspect of their cardioprotective action is enhancing endothelial health, pivotal in decelerating atherosclerosis progression. While GLP-1 primarily functions to lower blood glucose levels, both GLP-1 and GLP-1RAs have been shown in vitro and in vivo to reduce ROS and protect endothelial cells from oxidative damage caused by high glucose, fatty acids, cytokines, and hydrogen peroxide [107,112].

At the molecular level, activation of the GLP-1 receptor initiates intracellular signaling cascades involving cyclic AMP (cAMP), phosphoinositide 3-kinase (PI3K), and protein kinase C (PKC) pathways. These pathways subsequently activate the nuclear factor erythroid 2–related factor 2 (Nrf2) pathway, enhancing the cell’s antioxidant defenses [117]. Additionally, GLP-1RAs inhibit cellular senescence and protect endothelial cells from DNA damage and oxidative stress, as evidenced in human umbilical vein endothelial cells exposed to hydrogen peroxide [118].

In cardiac microvascular endothelial cells, hyperglycemia induces elevated ROS production, an increased apoptotic index, and the upregulation of NADPH oxidases such as p47phox and gp91phox. Treatment with GLP-1 at a concentration of 10 nmol/L mitigates these adverse effects by enhancing cAMP/PKA signaling and reducing Rho protein expression [119]. Tang et al. demonstrated that GLP-1RAs alleviate oxidative stress and endothelial injury by modulating the NF-κB signaling pathway in streptozotocin-induced diabetic rats [120]. Similarly, Bruen et al. found that liraglutide promotes endothelial cell survival through the activation of protein kinase A (PKA) and AMP-activated protein kinase (AMPK), as well as by increasing nitric oxide (NO) production in apolipoprotein E–deficient (ApoE^−/−^) mice [121].

Improvement in endothelial function, often measured by flow-mediated vasodilation (FMD), has been observed with GLP-1RA therapies such as exenatide and liraglutide in individuals with T2D and experimental ischemia-reperfusion injury [122,123,124]. Animal studies further confirm these findings, showing enhanced vasodilation, arterial relaxation, and coronary flow reserve with GLP-1RA treatment [125,126]. Additionally, the combination of dulaglutide and metformin has been demonstrated to reduce arterial stiffness more effectively than metformin alone [127].

Beyond endothelial protection, GLP-1RAs also mitigate systemic inflammation, thereby contributing to their cardioprotective effects. Clinical trials have reported significant reductions in inflammatory biomarkers, including CRP, TNF-α, IL-6, and interleukin-1β (IL-1β), following treatment with dulaglutide, liraglutide, and exenatide in patients with T2D [127,128,129,130,131]. In diabetic mice, exendin administration has been revealed to attenuate inflammatory responses by upregulating regulatory T cells [132] and diminishing levels of proinflammatory cytokines such as IL-1β and IL-18, adhesion molecules, and ROS [133,134].

High glucose levels induce excessive ROS production and the upregulation of NOX4 and TXNIP, activating the NLRP3 inflammasome. This activation results in pyroptosis and the maturation of IL-1β and IL-18, contributing to T2DM-related cardiovascular complications [134]. GLP-1RAs, particularly dulaglutide, have been shown to reduce NOX4 and TXNIP expression and suppress NLRP3 inflammasome activation by enhancing SIRT1 expression, thereby protecting against oxidative damage and inflammation [135]. Preclinical studies further demonstrate that GLP-1RAs decrease local inflammation, including reduced IL-1β expression in mouse aortas with lipopolysaccharide (LPS)-induced endotoxemia and X-ray-induced brain inflammation [136,137]. Additionally, GLP-1RAs suppress IL-1β expression in macrophages derived from endarterectomy samples, peripheral blood mononuclear cells (PBMCs), and human coronary artery endothelial cells cultured in high-glucose environments [121,130,138].

Mechanistically, the anti-inflammatory effects of GLP-1RAs involve inhibition of the NF-κB pathway, a key regulator of proinflammatory cytokines downstream of TNF-α. GLP-1RAs reduce NF-κB activation and the phosphorylation of its activators, c-Jun N-terminal kinase (JNK) and extracellular signal-regulated kinase (ERK1/2), as observed in plaque specimens, diabetic animal models, and human endothelial and macrophage cultures treated with GLP-1RAs [130,139,140,141,142]. Notably, the combined administration of a GLP-1 analog and fibroblast growth factor 21 (FGF21) has been shown to mitigate inflammation and atherosclerosis by modulating the Akt and ERK1/2 signaling pathways, suggesting potential clinical applications for managing diabetes-related vascular complications [143].

Additionally, GLP-1RAs help alleviate macrophage-driven insulin resistance by inhibiting the NF-κB pathway and reducing proinflammatory cytokine release, effects that depend on intact GLP-1 receptor expression [144,145,146]. Proinflammatory cytokines released by dysfunctional endothelium promote monocyte recruitment, a key process in atherogenesis. GLP-1RAs effectively suppress monocyte recruitment in preclinical models, as evidenced by semaglutide-reducing proinflammatory myeloid cells in the aortas of rabbits fed a high-cholesterol diet [147,148,149]. This suppression is mediated by reducing MCP-1, a chemokine that facilitates monocyte migration and endothelial permeability [150,151]. Liraglutide has similarly decreased MCP-1 expression in plasma, PBMCs, endarterectomy tissues, and high-risk mouse models [130,136,152].

Monocytes adhere to the vascular wall through endothelial adhesion molecules such as vascular cell adhesion molecule-1 (VCAM-1) and intercellular adhesion molecule-1 (ICAM-1). Exenatide reduces monocyte adhesion and infiltration into mouse aortic tissue by suppressing VCAM-1 and ICAM-1 expression [153,154]. These effects are absent in endothelial-specific GLP-1R knockout mice, underscoring the GLP-1R-dependent nature of these mechanisms [146].

Furthermore, GLP-1RAs influence macrophage polarization by promoting anti-inflammatory M2 phenotypes and reducing proinflammatory M1 macrophage activation. Liraglutide and exenatide decrease inducible nitric oxide synthase (iNOS) and STAT1 expression (M1 markers) while increasing arginase-1 (Arg-1) and STAT3 expression (M2 markers) in macrophages from ApoE^−/−^ mice, endotoxemic mice, and streptozotocin-induced diabetic rats [121,136,140].

Foam cell formation, a crucial step in plaque development, occurs when macrophages accumulate oxidized LDL (ox-LDL). Liraglutide and exenatide reduce cholesteryl ester accumulation in macrophages and reverse these effects when cAMP/PKA signaling is inhibited [148,149]. In ApoE^−/−^ mice, liraglutide infusion notably slows the expansion of atherosclerotic lesions marked by monocyte/macrophage infiltration within the aortic wall and reduces foam cell formation by downregulating cholesterol acyltransferase 1 (ACAT1) [148,149,155,156].

Moreover, GLP-1RAs have been shown to impact vascular smooth muscle cell (VSMC) proliferation and intimal thickening. Under hyperglycemia, lipotoxicity, and inflammation conditions, VSMCs shift from a contractile to a proliferative phenotype, leading to vascular wall thickening and arterial stenosis [125,157,158,159]. GLP-1RAs suppress VSMC proliferation through AMPK activation, inducing cell cycle arrest and promoting a contractile phenotype. Increased AMPK phosphorylation and upregulation of the cell cycle inhibitor p27 have been observed in VSMCs treated with liraglutide and exendin-4 [157,158,159]. Preclinical studies demonstrate that GLP-1RAs reduce VSMC accumulation and intimal thickness following arterial injury and decrease aortic hyperplasia in ApoE^−/−^ and insulin-resistant mice [125,158,160].

GLP-1RAs also contribute to plaque stability by reducing plaque formation and promoting stable plaque characteristics. In animal models, semaglutide has been shown to decrease fatty lesions in the aortic root and the iliac bifurcation [121,125,145,161], while lixisenatide leads to more stable plaques with smaller necrotic cores and reduced inflammation in ApoE^−/−^ mice and hyperlipidemic rabbits [162,163]. The STOP trial revealed that semaglutide converts noncalcified plaques to more stable calcified forms in T2D patients [164]. Additionally, GLP-1RAs lower matrix metalloproteinase (MMP) activity, particularly MMP-9, which weakens the fibrous cap and exacerbates plaque rupture, enhancing plaque stability [130,139,165].

Furthermore, GLP-1RAs mitigate thrombosis by reducing platelet activation and aggregation. Exenatide therapy increases cAMP and PKA activity, suppressing thrombus formation in hyperglycemic mice and human whole blood ex vivo [166,167]. GLP-1R knockout models confirm these antithrombotic effects, highlighting the role of G protein signaling in mediating these benefits [168].

Based on all the evidence, GLP-1 RAs offer multifaceted protective effects against atherosclerosis in diabetic patients through mechanisms that enhance endothelial function, reduce oxidative stress and inflammation, inhibit VSMC proliferation, stabilize plaques, and mitigate thrombosis. These comprehensive actions underscore the potential of GLP-1RAs in managing cardiovascular complications associated with diabetes.

Beyond GLP-1RAs, the role of glucose-dependent insulinotropic polypeptide (GIP) in atherogenesis is an active area of investigation. GIP receptors are expressed in endothelial and cardiac cells, and genetic studies have linked specific GIP receptor variants to increased cardiovascular risk associated with elevated GIP levels [169]. In vitro evidence indicates that GIP has dual roles in atherogenesis. On the one hand, GIP exerts anti-atherogenic effects by enhancing nitric oxide (NO) production and activating AMPK in vascular endothelial cells (VECs), which limits VSMC proliferation and reduces inflammation and foam cell formation in monocytes/macrophages and adipocytes [170]. On the other hand, GIP may promote atherogenesis by increasing endothelin-1 (ET-1) production in VECs, stimulating osteopontin synthesis in VSMCs, and aggravating inflammatory pathways in adipocytes [170]. Notably, reduced GIP receptor expression in animal models has demonstrated cardioprotective effects [171,172,173].

Preclinical studies in murine models have highlighted the anti-atherogenic properties of GIP. In apolipoprotein E knockout (ApoE^−/−^) mice, chronic infusion of active GIP (25 nmol/kg/day) for four weeks significantly attenuated aortic plaque development and intra-plaque macrophage accumulation without altering food intake, body weight, systolic blood pressure, or plasma glucose and lipid levels [174]. In contrast, infusion of inactive GIP showed no significant effects, underscoring the importance of GIP’s active form in mediating protective effects. Further analysis revealed that active GIP inhibited macrophage foam cell formation ex vivo by decreasing the expression of CD36 and acetyl-coenzyme A acetyltransferase-1 (ACAT1), which are involved in oxidized LDL uptake and intracellular cholesterol storage, respectively [171]. Similarly, in diabetic ApoE^−/−^ mice, active GIP infusion reduced aortic plaque formation, intra-plaque macrophage infiltration, and foam cell formation [172]. Additionally, GIP overexpression in non-diabetic ApoE^−/−^ mice stabilized aortic plaques by lowering macrophage content and increasing collagen deposition without promoting weight gain [173]. These findings suggest that pharmacological doses of GIP can confer atheroprotection without exacerbating obesity, though the effects of physiological doses of GIP on atherosclerosis require further investigation [170].

### 3.2. Clinical Insights into Glucagon-like Peptide-1 Receptor Agonists (GLP-1 RAs)

Over the past decade, clinical trials investigating GLP-1 receptor agonists have shifted their focus to evaluating their impact on major adverse cardiovascular events (MACE), beyond their known benefits on blood glucose control and weight reduction [175,176,177]. Dyslipidemia and blood pressure control are recognized as key risk factors contributing to the development of atherosclerosis and CVD in individuals with T2DM. Evidence from clinical trials has highlighted that GLP1Ras can [178] effectively address these risk factors, mainly by lowering low-density lipoprotein cholesterol (LDL-C) levels, thereby slowing the progression of atherosclerosis. Findings from a randomized controlled trial demonstrated that taspoglutide significantly reduced total cholesterol (TC), LDL-C, and triglycerides [178,179]. Furthermore, among newly diagnosed diabetes patients receiving standard statin therapy, the combination of liraglutide and metformin improved LDL distribution and lowered CRP levels, both associated with atherosclerosis [180]. Interestingly, research has also found that liraglutide at a daily dose of 1.2 mg alone provides more significant benefits for lipid metabolism and cardiovascular protection compared to its combination with metformin or metformin alone, even when glycemic control is comparable [181].

Additionally, in the LEAD series of studies, clinical evidence showed that a 26-week treatment with liraglutide resulted in a reduction in systolic blood pressure (SBP) [182]. Similarly, findings from the DURATION open-extension study demonstrated that exenatide improved blood pressure and lipid profiles in patients with T2DM over a period of seven years [179]. Consistent benefits were observed with dulaglutide in both the REWIND and AWARD-5 studies, where it effectively enhanced blood pressure and lipid levels [183,184]. The underlying mechanisms suggest that GLP-1 reduces blood pressure partly by promoting diuresis and influencing renal function [185,186]. Additionally, GLP-1RAs enhance systemic insulin sensitivity, which helps mitigate arterial hypertension in T2DM by lowering angiotensin II (Ang II) levels [187,188]

Furthermore, the treatment with liraglutide and exenatide was found to improve multiple cardiometabolic risk factors, including carotid intima-media thickness (cIMT), in patients suffering from both T2DM and metabolic syndrome (Mets) [189,190,191]. Moreover, the exploratory analyses of the eSTOP trial suggest that semaglutide might contribute to plaque stabilization by converting non-calcified plaques to calcified ones, a trend similar to that observed with statins and PCSK9 inhibitors [192].

Recent meta-analyses suggest these therapies are associated with a 12–14% decrease in MACE among individuals with T2D and pre-existing CV disease [193,194]. Key clinical trials, as detailed in Table 2, have been instrumental in elucidating these cardiovascular outcomes.

The ELIXA trial evaluated lixisenatide, a GLP-1RA, against a placebo in 6068 patients with T2DM who had recently experienced an acute coronary event. Over a median follow-up of 2.1 years, lixisenatide demonstrated non-inferiority to placebo regarding the primary composite endpoint of cardiovascular death, myocardial infarction (MI), stroke, or hospitalization for unstable angina, with MACE occurring in 13.4% of the lixisenatide group versus 13.2% of the placebo group (hazard ratio [HR] 1.02; 95% confidence interval [CI] 0.89–1.17; *p* < 0.001 for non-inferiority) [195].

In contrast, the LEADER trial assessed liraglutide in 9340 high-risk patients with T2DM, the majority of whom (81.3%) had established CVD. Over 3.8 years, liraglutide significantly reduced the incidence of the first occurrence of CV death, nonfatal MI, and nonfatal stroke compared to placebo (HR 0.87; 95% CI 0.78–0.97; *p* = 0.01 for superiority) [196]. Similarly, the SUSTAIN-6 trial investigated once-a-week semaglutide at doses of 0.5 mg and 1.0 mg in 3297 patients with T2DM with established CVD or CKD. Over 2.1 years, semaglutide was superior to placebo in reducing MACE (HR 0.74; 95% CI 0.58–0.95; *p* < 0.001 for non-inferiority and *p* = 0.02 for superiority) [197].

The EXSCEL trial expanded the evaluation to a larger cohort of 14,752 patients with T2DM, of whom 73.1% had previous CVD. This trial compared weekly exenatide to placebo over 3.2 years and found exenatide to be non-inferior to placebo in terms of safety (*p* < 0.001), but it did not achieve superiority in efficacy for reducing MACE (*p* = 0.06) [128,198,199,200,201].

In the Harmony Outcomes trial, albiglutide was tested against a placebo in 4731 patients with T2DM with established CVD over 1.6 years. Albiglutide was superior to placebo in reducing MACE, with the primary composite outcome occurring in 7% of the albiglutide group compared to 9% of the placebo group (HR 0.78; 95% CI 0.68–0.90; *p* < 0.001 for non-inferiority and *p* = 0.006 for superiority). Importantly, this cardiovascular benefit was achieved with minimal weight loss (<1 kg), suggesting that albiglutide’s cardioprotective effects are largely independent of weight reduction [199].

The REWIND trial evaluated dulaglutide in 9901 patients with T2DM, of whom 31.5% had a history of CVD. Over 5.4 years, dulaglutide was superior to placebo in reducing MACE (HR 0.88; 95% CI 0.79–0.99; *p* = 0.026) [183]. In the PIONEER 6 trial, oral semaglutide was compared to placebo in 3183 patients with T2DM, 84.7% aged 50 or older with CVD or chronic kidney disease. Over a median follow-up of 15.9 months, oral semaglutide was non-inferior to placebo for MACE (HR 0.79; 95% CI 0.57–1.11; *p* < 0.001) but did not demonstrate superiority (*p* = 0.17) [201].

Extending the benefits of GLP-1RAs beyond diabetes management, the SELECT trial investigated semaglutide at a dose of 2.4 mg weekly in 17,604 overweight or obese individuals without diabetes but with established ASCVD. Over a median follow-up of 3.3 years, semaglutide resulted in a 20% relative reduction in MACE compared to placebo (HR 0.80; 95% CI 0.72–0.90; *p* < 0.001), with primary endpoints occurring in 6.5% of the semaglutide group versus 8.0% of the placebo group [128]. These findings led to FDA approval in March 2024 for semaglutide to reduce cardiovascular events in individuals with overweight or obesity and established CVD.

Additionally, GLP-1RAs offer other cardiometabolic benefits, such as improved symptoms and physical functioning in heart failure with preserved ejection fraction (HFpEF), as demonstrated by the STEP-HFpEF trial [202], and the 24% reduced risk of major kidney adverse effects and 18% reduced risk of MACE reported recently in the results of the FLOW study [203]. Upcoming trials, including SYNERGY-NASH, STRIDE, SURMOUNT-OSA, SUMMIT, SURMOUNT-MMO, and EVOKE, aim to explore the broader impacts of both monoincretin and dual-incretin agonists on various cardiometabolic and neurological conditions. Despite the clear cardiovascular advantages, long-term adherence to GLP-1RA therapy is essential to maintain weight loss and cardiometabolic benefits, as some studies have observed significant rebound weight gain upon discontinuation.

In summary, GLP-1 RAs demonstrate a robust and multifaceted approach in combating atherosclerosis by targeting critical stages of plaque development. Their ability to enhance endothelial function, reduce inflammation, improve lipid profiles, prevent foam cell formation, inhibit smooth muscle cell proliferation, stabilize plaques, and mitigate thrombosis underscores their significant potential as comprehensive therapeutic agents for cardiovascular protection in patients with T2D and associated vascular risks. These conclusions are strongly supported by valuable evidence from preclinical research and numerous clinical trials and studies, which consistently highlight the cardiovascular benefits of GLP-1 RAs beyond their roles in glycemic control and weight management. Consequently, GLP-1 RAs are invaluable in managing and preventing atherosclerotic complications, offering substantial cardiovascular protection for individuals with type 2 diabetes.

## 4. Evaluating the Impact of SGLT2 Inhibitors on Atherosclerosis and Cardiovascular Outcomes in Type 2 Diabetes

SGLT2 inhibitors were initially developed as targeted therapies against glucose transporter-2, facilitating the excretion of intracellularly accumulated glucose from tubular cells into the interstitial fluid and peritubular capillaries. Beyond their primary function of reducing glucose reabsorption from urine and reducing glucose levels for individuals with T2DM, SGLT2 inhibitors have demonstrated additional advantageous pleotropic effects. While some of these benefits directly relate to their action on renal tubules, SGLT2 inhibitors also exert effects independently of this pathway. This is supported by experimental evidence showing the cardioprotective effects of empagliflozin in mice lacking renal SGLT2 expression [204]. Regarding their anti-atherosclerotic effects, SGLT2 inhibitors may directly benefit atherosclerotic plaques [205]. They can decrease the activity of the NLRP3 inflammasome, beneficially modulate macrophage polarization towards the anti-inflammatory M2 phenotype, and reduce vessel wall infiltration [206].

Additionally, the amelioration of glucotoxicity, associated with reduced plasma glucose concentrations, may explain the increased insulin sensitivity observed following SGLT2 inhibitor use. Furthermore, SGLT2 inhibitors might influence atherosclerotic risk through the effects on gut microbiota [207].

### 4.1. Preclinical Evidence

#### 4.1.1. Effects of SGLT2 Inhibitors on Atherosclerosis and Endothelial Dysfunction

SGLT2 inhibitors have emerged as promising agents in managing atherosclerosis and endothelial dysfunction, particularly in diabetic models. Preclinical studies elucidate multiple mechanisms through which SGLT2 inhibitors exert their protective effects.

Canagliflozin has been demonstrated to reduce the progression of atherosclerosis by attenuating inflammatory processes in diabetic atherosclerosis-prone apolipoprotein E-deficient (ApoE^−/−^) mice [208]. Similarly, it exhibits antioxidant properties in high-fat diet diabetic ApoE^−/−^ mice by decreasing the release of interleukin-1 beta (IL-1β) from macrophages [209]. The antioxidant effects of dapagliflozin and empagliflozin contribute to the preserving endothelial function by mitigating ROS and reducing oxidative stress [210]. In addition, dapagliflozin improves endothelial function by decreasing the expression of vascular adhesion molecules, inhibiting NF-κB activation, and reducing macrophage infiltration into the vessel wall [211,212]. Moreover, the empagliflozin attenuates atherogenesis and endothelial dysfunction in diabetic ApoE^−/−^ mice by modulating the expression of MCP-1, CD68, and subunits of NADPH oxidase in macrophages [213]. Similarly, dapagliflozin-treated diabetic mice exhibited enhanced endothelial and VSMC function and reduced arterial stiffness [211]. In murine models with early CV dysfunction and mild diabetes, empagliflozin increased coronary flow velocity reserve, thereby improving coronary microvascular function [214]. Additionally, empagliflozin restored the integrity of the endothelial glycocalyx, a critical component of vascular health [215].

Empagliflozin also enhanced endothelium-dependent relaxation in streptozotocin-induced diabetic rats [216] and restored endothelial function in genetically diabetic db/db mice [217].

In addition, numerous investigations have shown that SGLT2 inhibitors (dapagliflozin, empagliflozin, canagliflozin, luseogliflozin, and ipragliflozin) help protect against atherosclerosis by improving metabolic parameters. In addition, dapagliflozin was found to decrease fasting blood glucose (FBG), TC, and triglycerides (TG) [218], as well as body weight and glycosylated hemoglobin (HbA1c) [219], in both STZ-induced diabetic ApoE^−/−^ mice and STZ-induced diabetic Ldlr^−/−^ mice [220]. Meanwhile, empagliflozin lowered FBG, TC, heart rate, blood pressure (BP), TG, LDL [221] urinary microalbumin, body weight, and fat mass [221,222] in ApoE^−/−^ mice fed a high-fat diet (HFD). It also reduced body weight and TG in STZ-induced diabetic ApoE^−/−^ mice [213].

Collectively, these preclinical findings demonstrate that SGLT2 inhibitors confer atheroprotective effects through anti-inflammatory actions, a reduction in oxidative stress, enhancement of the endothelial function, modulation of key molecular signaling pathways, and improvement in VSMC function. These multifaceted mechanisms underscore the therapeutic potential of SGLT2i in managing atherosclerosis and endothelial dysfunction in diabetic populations.

#### 4.1.2. Effects on Microbiota

Seeking novel therapeutic strategies for T2DM reveals the complex relationship between SGLT2 inhibitors and the gut microbiota as a promising area for exploration. Indeed, SGLT2 inhibitors offer benefits in atherosclerosis management in T2DM through their effects on the gut bacteria. Preliminary research indicates that various SGLT2 inhibitors can induce beneficial changes in gut microbiota composition, enhancing their therapeutic effects. However, clinical studies have shown inconsistent findings, pointing to the complexity of this relationship.

Nevertheless, SGLT2 inhibitors induced changes in the composition of the gut microbiota in animal studies. Empagliflozin has also been shown to positively alter the gut microbiota in T2DM mice by increasing SCFA- and reducing LPS-producing bacteria [223]. This change was associated with an amelioration of diabetic nephropathy through an increase in SCFA production in dapagliflozin-treated T2DM mice [223]. In fact, in dapagliflozin-treated diabetic mice fed with butyrate, the microbiota changes consisted of a shift toward a more favorable decreased Firmicutes to Bacteroidetes ratio, reductions in Adlercreutzia and Alistipes spp., and an increase in Streptococcus spp [224], as well as an abundance of Akkermansia muciniphila with decreased Oscillospira spp [220]. A recent study in db/db mice treated with dapagliflozin demonstrated a dynamic improvement in the gut microbiota over time [225]. Similar results were found in animal models with canagliflozin, consisting of a significantly enhanced production of SCFAs and reduced plasma levels of p-cresyl sulfate and indoxyl sulfate in the intestine [226].

### 4.2. Clinical Studies Investigating the Effects of SGLT2 Inhibitors on Atherosclerosis Mechanisms in Type 2 Diabetes

#### 4.2.1. The Effect of SGLT2 Inhibitors on Inflammatory Markers

The effect of SGLT2 inhibitors on inflammatory markers has been the subject of extensive investigation in clinical trials. These drugs have been observed to influence various inflammatory markers, including CRP and IL-6, with clinical studies yielding varying results. While some studies report significant reductions in these markers with SGLT2i treatment, others show minimal or no change, reflecting the complexity and variability of their anti-inflammatory effects.

CRP: Clinical studies examining the effect of SGLT2 inhibitors on CRP have yielded inconsistent findings. Regarding canagliflozin, an open-label study in a small group of Japanese patients with T2DM with stable chronic heart failure reported a significant reduction in CRP at 3, 6, and 12 months [227]. Similarly, in newly diagnosed people with T2DM, a 12-week course of canagliflozin (100 mg once daily) also lowered CRP [228]. In contrast, no change was observed after 12 weeks of treatment (100 mg once daily) in 12 Japanese subjects with T2DM [229], and the post-doc exploratory analysis of the CANTATA-SU study revealed only a non-significant decrease [230].

Evidence for dapagliflozin is equally mixed. Namely, a 6-week regimen (10 mg/day) reduced CRP from baseline, although this difference was not statistically significant compared to placebo [231]. Meanwhile, in a 12-week trial of dapagliflozin (10 mg/day) added to metformin, CRP levels were significantly decreased in 59 subjects with T2DM [232]. Conversely, in a 24-week open-label, uncontrolled pilot study (5 mg/day) in 11 patients with T2DM with non-alcoholic steatohepatitis there were almost no changes in CRP [233], while in a separate 12-week intervention (10 mg/day) in 36 subjects with T2DM, there was an unexpected increase in median CRP [234]. Furthermore, dapagliflozin did not differ from glibenclamide in patients with T2DM with subclinical carotid atherosclerotic disease [235] or vildagliptin in patients with T2DM with coronary artery disease [236].

Studies on empagliflozin have produced similarly variable outcomes. In a 12-month randomized, open-label, prospective trial of 51 subjects with T2DM, empagliflozin yielded a 54% reduction in hs-CRP compared with placebo [237], and a 6-month intervention in patients with T2DM with coronary artery disease also reported a significant decrease in CRP [238]. However, a 6-week study of empagliflozin (25 mgday) in 58 subjects with T2DM showed no difference compared to placebo [239], and the post hoc analysis of the Empagliflozin in Heart Failure Patients with Reduced Ejection Fraction (Empire HF) trial that found no change in CRP [240]. In addition, in a prospective RCT involving diabetic and non-diabetic CKD populations, no clinically meaningful effect was observed [241]. These findings highlight the variability in the effects of SGLT2 inhibitors on CRP levels across different studies, which may be due to factors such as patient population, treatment duration, and study design, underlining the need for further investigation into the mechanisms underlying these discrepancies.

IL-6 levels: Conflicting data have been reported regarding the impact of SGLT2 inhibitors on IL-6 levels. In the case of canagliflozin, no significant changes were observed after 24 weeks of treatment (100 mg/day) [242]. However, a higher dose (300 mg/day) administered for 52 weeks led to a 22% reduction in IL-6 among 100 patients with T2DM [230]. However, the evidence for dapagliflozin is similarly inconsistent, with certain studies finding no change in IL-6 levels [235,243], while an 8-week randomized, double-blind, placebo-controlled study demonstrated a significant reduction [242]. As for empagliflozin, this treatment did not significantly alter IL-6 levels in subjects with T2DM [244], although a distinct trial in patients with T2DM with reduced ejection fraction (HFrEF) reported a significant decrease [245].

TNF-α: Only a limited number of investigations have focused on the effect of SGLT2 inhibitors on serum TNF-α levels, comprising three placebo-controlled studies [218,243,244,246] and four comparisons with other glucose-lowering agents [22,230,235,236]. None reported a significant impact on TNF-α. Additionally, conflicting results have emerged regarding other inflammatory markers. Overall, although specific biomarkers appear to be modulated under SGLT2 inhibitor therapy, current evidence does not conclusively support a robust effect on circulating CRP, IL-6, or TNF-α levels, leaving the drugs’ anti-inflammatory potential uncertain. Further well-designed clinical trials with larger cohorts of patients with T2DM are warranted to elucidate any definitive anti-inflammatory benefits.

#### 4.2.2. Effects on Insulin Resistance and Metabolic Memory

Treatment with SGLT2 inhibitors was observed to modestly improve insulin sensitivity by ∼25% to 30% in diabetic animal experimental models [247] and clinical T2DM studies [248] within 2 weeks of treatment initiation. In a cross-sectional study evaluating the effect of SGLT2 inhibitors on insulin secretion and resistance, SGLT2 inhibitors blunted glucagon-induced insulin secretion in β-cells and improved insulin resistance [249]. Interestingly, this effect was independent of visceral fat mass. The body weight reduction caused by SGLT2i may have been related to fat utilization increase and adipose tissue mass reduction associated with obesity-induced insulin resistance attenuation. In a cohort study, it was shown that the early introduction of SGLT-2i was able to eliminate the association between poor glycemic control in the first two years after T2D diagnosis and the later development of CVD, measured as a composite of myocardial infarction, stroke, coronary or peripheral bypass, and coronary or peripheral revascularization. This effect was not influenced by glycemic control. Although these findings require validation in prospective cohorts, if they are replicated in additional studies, they support the notion that SGLT2 inhibitors act as disease-modifying drugs, with the potential to reduce the harmful long-term effects associated with an inadequate glycemic control in the early years following T2D diagnosis [250].

#### 4.2.3. Effects of SGLT2 Inhibitors on Metabolite Excretion, Lipid Profiles, and Gut Microbiota in Type 2 Diabetes and Atherosclerosis

SGLT2 inhibitors interfere with the excretion of metabolites that have a role in cardiovascular system, such as purine metabolites. The uricosuric effects of SGLT2 inhibitors have been described in experimental and clinical studies, showing the potential role of the urate transporter URAT1 (urate transporter 1). An in-depth study using proteomics, phosphoproteomics, and metabolomics analysis after 1 week of SGLT2 inhibitor treatment in diabetic as well as nondiabetic mice showed evidence of a direct interaction of SGLT2 inhibitors with URAT1 (Slc22a12), as well as a reduced protein expression of URAT1 and reduced phosphorylation at S534 by SGLT2 inhibitors [251].

SGLT2 inhibitors also lead to beneficial lipid changes toward a less atherogenic profile, characterized by a modest decrease in plasma TG, an increase in high-density lipoprotein cholesterol (HDL-C), and a modest increase in LDL, with a potential mechanism being related to a decreased LDL clearance, a greater lipolysis of triglyceride-rich lipoproteins [252], and an energy metabolism switch toward lipid utilization [253].

Despite the beneficial impact yielded in preclinical studies, there are limited clinical studies on the impact of SGLT2 inhibitors on the fecal microbiome, and they have produced inconsistent results. A study conducted with Japanese patients with T2DM found that treatment with an SGLT2 inhibitor (luseogliflozin or dapagliflozin) was associated with an overall increase in the prevalence of balance-regulating bacteria, including SCFA-producing bacteria [254]. In a randomized, open-label trial, conducted in 76 previously treatment-naïve patients with T2DM, empagliflozin use for three months produced substantial beneficial alterations in the gut microbiota, on top of improvements in glucose metabolism and a reduction in cardiovascular risk [255]. These changes consisted of a decrease in harmful bacteria and an increase in beneficial SCFA-producing bacteria. In contrast, a double-blind randomized study in 44 patients with T2DM, failed to show any significant impact of dapagliflozin on the gut microbial composition [256].

In the in-depth study of proteomics, phosphoproteomics, and metabolomics analysis mentioned above, SGLT2i particularly affected the gut microbiome, with a lower relative number of bacteria taxa capable of fermenting tryptophan and phenylalanine to cardiovascular uremic toxins, resulting in lower plasma levels of these compounds (including p-cresol sulfate). The reduced microbiome formation of uremic toxins, their decreased body exposure, and the need for renal detoxification, combined with the direct effects of SGLT2i in the kidney, provides a metabolic foundation for kidney and cardiovascular protection [251].

### 4.3. Clinical Insights into the Role of SGLT2 Inhibitors in Managing Atherosclerosis and Cardiovascular Risks in T2D

SGLT2 inhibitors reduce ASCVD events in patients with prior ASCVD and type 2 diabetes. In a systematic review and meta-analysis of randomized, placebo-controlled trials of cardiovascular outcomes in a total of 34,322 patients with T2DM, SGLT2 inhibitors showed moderate benefits on atherosclerotic major adverse cardiovascular events with established atherosclerotic cardiovascular disease [257]. This effect was also confirmed in subjects without established ASCVD. In a meta-analysis including selected large-scale cardiovascular outcome randomized placebo-controlled trials or their prespecified subgroup analyses, evaluating SGLT2 inhibitors for primary prevention of atherosclerotic CVD, SGLT2 inhibitors significantly reduced atherosclerotic MACEs in patients with both T2DM and CKD without established ASCVD [258].

A meta-analysis of the real-world effect of SGLT-2 inhibitors on CV outcomes in patients with T2DM, including fourteen trials enrolling a total of 3,157,259 patients, showed a predominant impact of SGLT2 inhibitors on CV outcomes. SGLT-2i did show sustained benefits on reducing MACE, MI, and stroke, independent of a history of usage of GLP-1RA and/or statins and /or metformin [259].

#### Cardiovascular Effects of SGLT2 Inhibitors

Overall, SGLT2 inhibitor treatment in various clinical studies demonstrated beneficial cardiovascular outcomes in patients with T2DM. In addition to the insulin-independent reduction in high glucose, SGLT2 inhibitors improve risk factors for CVD, such as body weight, systolic blood pressure, uric acid, and lipid profile, and potentially protect against the pathogenesis of atherosclerosis [260].

Canagliflozin substantially reduced MACE (nonfatal MI, nonfatal stroke, or CV death) in patients with different body mass indices. It also showed a reduction in MACE in the primary and secondary prevention of CV events. In a post hoc analysis of pooled data from a randomized, double-blind, placebo-controlled study that assessed canagliflozin in patients with T2DM (*n* = 2313) and a 6-week study involving patients with T2DM and hypertension (*n* = 169) [261], the participants received 100 or 300 mg of canagliflozin as monotherapy or as add-on therapy. Canagliflozin reduced pulse and mean arterial pressure compared to the placebo at week 26. These beneficial effects are likely to translate into reduced arterial resistance, arterial stiffness, and improved blood flow. The effects of canagliflozin for the primary and secondary prevention of CVD in individuals with and without prior CVD were evaluated in the Canagliflozin Cardiovascular Assessment Study (CANVAS) [262] over a follow-up period of 3.5 years. It was observed that canagliflozin, in comparison to placebo, homogenously reduced nonfatal MI, nonfatal stroke, and CV death among participants in the primary and secondary prevention groups, with a higher absolute risk reduction in the secondary prevention group [262]. When participants were categorized according to BMI levels, canagliflozin substantially reduced the risk of composite CV events compared to placebo across all BMI levels [263]. However, the study could not draw clear conclusions regarding the effects of canagliflozin in patients with lean bodies [263].

Dapagliflozin has not demonstrated a significant reduction in MACE in clinical studies. In the DECLARE-TIMI 58 trial, which included patients with T2DM at elevated risk for ASCVD, dapagliflozin was evaluated for its impact on CV outcomes [264]. After a median follow-up of 4.2 years, dapagliflozin was found to be non-inferior to placebo concerning MACE, which encompassed MI, ischemic stroke, and CV death. The absence of a significant reduction in MACE remained consistent across various age groups [265]. Additionally, a secondary analysis of the DECLARE-TIMI 58 trial assessed the effects of dapagliflozin on CV outcomes based on baseline kidney function and albuminuria in patients with T2DM [266]. The analysis revealed a more consistent relative risk reduction in cardiovascular events with dapagliflozin use, irrespective of baseline estimated glomerular filtration rate (eGFR) and the degree of albuminuria. Notably, patients exhibiting more pronounced markers of CKD experienced a substantially greater absolute risk reduction in composite CV mortality [R28]. These findings suggest that while dapagliflozin does not significantly lower MACE overall, its CV benefits may be more evident in specific subpopulations with advanced CKD [266].

Empagliflozin has been shown to reduce the risk of MACE in patients with established CVD and diverse CV risk profiles. In a post hoc analysis of the EMPA-REG OUTCOME trial, which included 7020 individuals with T2DM randomized to receive empagliflozin or placebo, empagliflozin significantly lowered the risk of 3-point MACE (nonfatal myocardial infarction, nonfatal stroke, or cardiovascular death) irrespective of the number of CV risk factors controlled at baseline [267]. However, these results may not be generalizable to patients with T2DM without established ASCVD [267]. Additionally, another study within the same trial population examined the effects of empagliflozin on cardiovascular events in patients with T2DM with a history of coronary artery bypass grafting (CABG) surgery. Among the 7020 participants, 25% of those receiving empagliflozin and 24% of those receiving placebo had undergone CABG, and no significant difference in the risk of myocardial infarction or stroke was observed between the two groups [268]. Further analysis using latent class analysis identified three phenotypic groups within the EMPA-REG OUTCOME trial: younger patients with a shorter duration of T2DM and higher glomerular filtration rates, predominantly female patients without coronary artery disease, and older adults with advanced coronary artery disease and multiple comorbidities. Empagliflozin consistently reduced the risk of cardiovascular death across all phenotypic groups compared to placebo [269]. These findings underscore the efficacy of empagliflozin in reducing cardiovascular mortality across varied patient profiles within the T2DM population. Table 3 presents the major adverse cardiovascular event outcomes in key SGLT2 inhibitor trials for type 2 diabetes.

Sotagliflozin has also been studied for its cardiovascular effects in patients with T2DM and CKD with or without albuminuria. In a short-term (15.9 months) study, sotagliflozin demonstrated a significant reduction in the risk of composite CV endpoint (death from CV causes, nonfatal MI, or nonfatal stroke) [270]. Treatment with sotagliflozin reduced the risk of the original coprimary end point (the first occurrence of death from CV causes, nonfatal MI, or nonfatal stroke) compared to placebo [270].

Ertugliflozin was assessed in a multicenter study involving patients with T2DM and established CVD, including coronary artery disease and peripheral artery disease (PAD). In this study, participants were randomized in a 1:1:1 ratio to receive either ertugliflozin 5 mg, ertugliflozin 15 mg, or placebo, and were followed for an average duration of 3.5 years [271]. The results demonstrate that ertugliflozin treatment was non-inferior to placebo regarding MACE, which comprised nonfatal MI, nonfatal stroke, and death from CV causes. These findings indicate that ertugliflozin does not increase the risk of MACE in patients with T2DM with established CVD compared to the placebo, supporting its safety profile in this high-risk population.

To summarize, clinical trials have demonstrated that SGLT2 inhibitors significantly improve cardiovascular outcomes in patients with T2DM by reducing major adverse cardiovascular events, lowering blood pressure, and favorably modifying lipid profiles. Notably, canagliflozin and empagliflozin have shown substantial efficacy in both the primary and secondary prevention of atherosclerotic cardiovascular disease and possess uricosuric effects that enhance their cardioprotective mechanisms. Although their impact on the gut microbiota varies and evidence of their anti-inflammatory effects remains inconsistent, SGLT2 inhibitors remain a valuable therapeutic option for managing atherosclerosis and improving cardiovascular health in patients with T2DM, warranting further research to fully elucidate their multifaceted benefits.

## 5. Nonsteroidal Mineralocorticoid Receptor Antagonists (nsMRA) in Diabetic Atherosclerosis

Elevated activation of both systemic and local renin–angiotensin–aldosterone systems (RAAS) is frequently observed in individuals with obesity and insulin resistance, significantly contributing to the development of T2DM and its associated cardio-renal complications [272,273,274]. Aldosterone, a pivotal steroid hormone, regulates blood pressure and maintains CV homeostasis by acting on renal and vascular mineralocorticoid receptors (MRs). This interaction primarily facilitates sodium reabsorption and potassium excretion in the distal tubules and collecting ducts of nephrons [273,275]. MRs are extensively expressed in various cell types, including endothelial cells, vascular smooth muscle cells, adipocytes, immune cells, skeletal muscle cells, and cardiomyocytes [273].

The disruption of aldosterone regulation, as seen in obesity and diabetes mellitus, is detrimental, enabling the progression of CV and kidney diseases [273]. Diabetic atherosclerosis, which is markedly exacerbated by CKD, is driven by endothelial dysfunction and increased inflammatory and oxidative stress. The mineralocorticoid receptor (MR) regulates vascular function and CV risk factors in these processes. MR activation is associated with enhanced vascular oxidative stress, inflammation, cellular proliferation and migration, vasoconstriction, vascular remodeling, and fibrosis, as demonstrated in studies involving the overexpression or deletion of MRs in endothelial, vascular smooth muscle, and cardiomyocytes in both human and animal models [276,277].

### Therapeutic Implications of MRA in Diabetic Atherosclerosis and Cardiovascular Disease: From Preclinical Research to Clinical Trials

Previously, an MRA, spironolactone, was shown to improve endothelial dysfunction in patients with hypertension [278]. However, the effect was not seen in patients with T2DM [279]. Treatment of patients with heart failure as a complication of an acute myocardial infarction with a selective MRA eplerenone resulted in improved endothelial function [280]. Experimental studies in both hypertensive and atherosclerotic animal models revealed that eplerenone improved endothelial function by increasing bioavailable NO, reducing oxidant stress, and restricting vascular remodeling [281,282].

MRA has been shown in several studies to reduce atherosclerosis in animal models, as seen by decreased proinflammatory cytokines in plaques, enhanced M2 markers, smooth muscle proliferation, and decreased inflammatory cell infiltration [283]. The important effects of eplerenone in lowering aortic intimal volume (intravascular ultrasonography) and enhancing acetylcholine-induced vasorelaxation have also been shown in animal studies on monkeys [284]. In response to Ang II, chimeric ApoE^−/−^ mice with myeloid MRKO also showed decreased atherogenesis. This effect was partially mediated by increased cholesterol efflux and decreased foam cell production [285].

Although the benefit of using MRA in secondary prevention in patients with heart failure symptoms and post-myocardial infarction is well established, there is evidence that MRA may be helpful when used early after MI in individuals with STEMI and left ventricular dysfunction [286]. In diabetic patients, eplerenone increased the ventricular myocardial perfusion reserve without increasing brachial artery vascular reactivity [287]. Even though, contrary to spironolactone, eplerenone did not lead to a further reduction in endothelial function in patients with diabetes, this subgroup of patients present a need for further drug development and better therapeutic approach to improving endothelial dysfunction and, thus, cardiovascular outcome.

A recent meta-analysis indicated that nonsteroidal MRAs had a beneficial effect on reducing the risk of the composite kidney and cardiovascular outcomes, and all-cause mortality [288]. Moreover, nonsteroidal MRAs were associated with benefits such as lowering blood pressure and improved proteinuria remission. The new nsMRA, finerenone, lowered the risk of kidney and cardiovascular outcomes in individuals with CKD and type 2 diabetes, according to two recent randomized trials: FIDELIO-DKD and FIGARO-DKD [289]. When compared to a placebo, finerenone improved cardiovascular outcomes in patients with type 2 diabetes and stage 2 to 4 CKD who had moderately elevated albuminuria and in patients with stage 1 or 2 CKD who had substantially elevated albuminuria [290]. Finerenone decreased the composite cardiovascular outcomes of hospitalization for heart failure, nonfatal myocardial infarction, nonfatal stroke, and mortality from cardiovascular causes over a median follow-up of 3.0 years, according to the FIDELITY study [289]. Unlike existing treatments, finerenone may lower fibrosis and inflammation, improving the care of these patients [291].

With almost 6000 patients with ASCVD and 7000 without, the secondary analyses of FIDELITY show that the advantages of finerenone are unaffected by the presence of ASCVD at baseline [292]. These findings imply that the protection against heart failure (HF) provided by finerenone is not dependent on a history of ASCVD, of which coronary artery disease is the primary cause of HF. This suggests that finerenone addresses some of the underlying pathogenetic mechanisms that cause HF in patients with T2D and CKD [292].

These medications may help lessen the rapid vascular aging observed in diabetic patients with CKD by avoiding aldosterone-induced damage to the vasculature, which could enhance outcomes and the quality of life. Hence, nonsteroidal MRAs present a prospective addition to the treatment toolkit for diabetic atherosclerosis, especially in patients who are at high cardiovascular risk because of coexisting kidney disease.

## 6. A Four-Pillared Therapeutic Strategy in Diabetic Kidney Disease: Integrating RAS Blockade, SGLT2 Inhibitors, GLP-1 RAs, and ns-MRAs for Optimal Cardiorenal Protection

A growing consensus endorses a “pillared” approach to diabetic kidney disease (DKD) management, wherein SGLT2 inhibitors, GLP-1 receptor agonists, and nonsteroidal mineralocorticoid receptor antagonists (ns-MRAs) are added to RAS blockade in patients with albuminuria. This strategy aims to address the interlinked mechanisms of metabolic dysregulation, hemodynamic stress, and inflammation that underlie DKD and its heightened atherosclerotic risk. Accordingly, major guidelines (KDIGO, ADA/EASD, and NICE) have embraced a stepwise pathway in which SGLT2 inhibitors and RAS blockade serve as first-line therapy, followed by GLP-1 receptor agonists and ns-MRAs to further reduce cardiovascular and renal complications [293,294,295,296]. The evidence from clinical trials and observational studies suggests that combining SGLT2is and GLP-1 RAs provides additional cardiovascular and renal benefits beyond glucose lowering [297,298,299,300,301,302,303,304,305]. A meta-analysis of five GLP-1RA cardiovascular outcome trials (ELIXA, LEADER, SUSTAIN-6, EXCEL, Harmony Outcomes) and three SGLT2i trials (EMPA-REG OUTCOME, CANVAS Program, DECLARE-TIMI 58) encompassing 77,242 participants demonstrated that both drug classes significantly reduced MACEs, with GLP-1 RAs uniquely lowering stroke incidence [293]. Multiple studies, including randomized controlled trials [306,307,308], non-randomized investigations [297,298,299,300], real-world analyses [309,310], and post hoc evaluations of cardiovascular outcomes trials [311,312,313,314,315], further support their benefits in glycemic control, weight reduction, and blood pressure management [307,316,317,318]. Moreover, the combination of GLP-1 receptor agonists, SGLT2 inhibitors, and aggressive lipid-lowering therapy—including statins, ezetimibe, bempedoic acid, and PCSK9 inhibitors—could be utilized to effectively reduce LDL levels and promote the regression of atherosclerotic plaques in coronary arteries [319].

Beyond cardiovascular and renal protection, diabetes-related foot ulceration (DFU) represents a severe complication intricately linked to systemic inflammation and infection, supporting the concept of a “cardio–renal–metabolic–foot” association. This bidirectional relationship implies that cardiovascular and kidney dysfunction not only contribute to but also result from DFU progression. Given their vascular and metabolic benefits, SGLT2is and GLP-1 RAs may mitigate DFU risk by enhancing endothelial function, reducing inflammation, and improving overall metabolic stability. A holistic, multidisciplinary approach integrating cardiovascular and renal care is essential in optimizing DFU management and reducing long-term complications [320,321].

Although a dedicated randomized controlled trial explicitly evaluating the combined impact of SGLT2is and GLP-1 RAs on cardiovascular and renal protection is still needed, the ongoing PRECIDENTD trial (NCT05390892) aiming to clarify these effects in 9000 high-risk individuals with type 2 diabetes, further refined their clinical utility in improving outcomes in this population. Meanwhile, pooled data from CANVAS, CREDENCE, FIDELIO-DKD, FIGARO-DKD, and eight GLP-1RA trials show that adding finerenone to SGLT2i and GLP-1 RA therapy, compared with standard management, yields a hazard ratio of 0.65 (95% CI, 0.55–0.76) for MACE, corresponding to a 4.4% absolute risk reduction (95% CI, 3.0–5.7) over three years and a number needed to treat of 23 (95% CI, 18–33). In a 50-year-old patient, this triple combination extended MACE-free survival from 17.9 to 21.1 years—a gain of 3.2 years (95% CI, 2.1–4.3) [322]. These findings underscore the promise of a fully integrated regimen to meaningfully curtail atherosclerotic complications, extend both event-free and overall survival for individuals with type 2 diabetes and CKD, and preserve kidney health in at-risk individuals [323].

**Figure 2:**
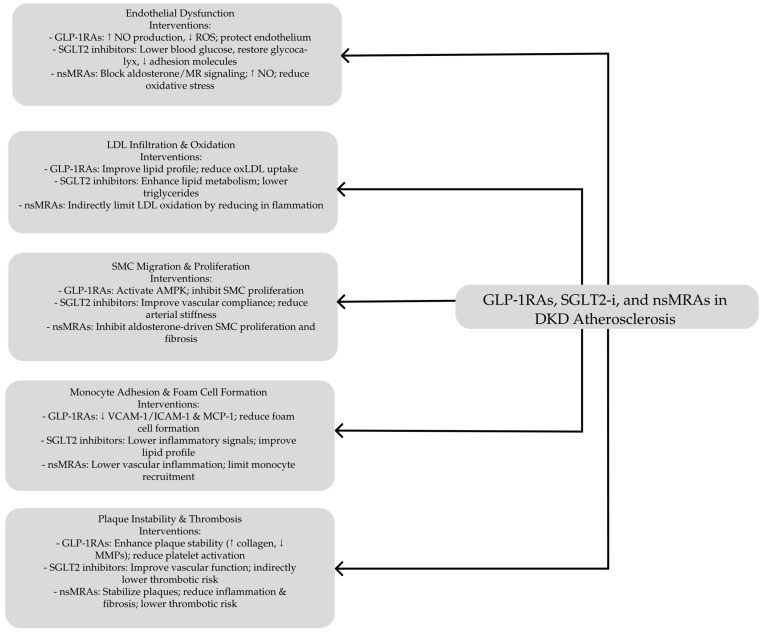
Targeting diabetic atherosclerosis: the role of GLP-1 receptor agonists, SGLT2 inhibitors, and nonsteroidal MRAs in vascular protection and disease modulation. DKD, Diabetic Kidney Disease; GLP-1RAs, Glucagon-Like Peptide-1 Receptor Agonists; SGLT2-i, Sodium-Glucose Cotransporter 2 Inhibitors; nsMRAs, Nonsteroidal Mineralocorticoid Receptor Antagonists; NO, Nitric Oxide; ROS, Reactive Oxygen Species; AMPK, 5′ AMP-Activated Protein Kinase; LDL, Low-Density Lipoprotein; SMC, Smooth Muscle Cell; VCAM-1, Vascular Cell Adhesion Molecule-1; ICAM-1, Intercellular Adhesion Molecule-1; MCP-1, Monocyte Chemoattractant Protein-1; MMPs, Matrix Metalloproteinases. ↓ deacrease; ↑ increase.

Recent clinical guidelines advocate for an early adoption of novel therapies to mitigate cardiovascular risk in patients with diabetes and CKD, and observational data suggest that a fully implemented multi-modal regimen can significantly improve cardiorenal outcomes in these individuals. However, real-world evidence shows that only a minority of patients with type 2 diabetes and ASCVD receive these guideline-endorsed therapies, with substantial disparities highlighting a treatment-risk paradox wherein higher-risk individuals are less likely to receive optimal care. Delays in treatment initiation—driven by clinical inertia, challenges in dose titration due to side effects, deferral in patients with complex comorbidities, high discontinuation rates, prohibitive drug costs, and limited insurance coverage—further reduce the effectiveness of these innovations [324,325]. In contrast, the center-based CINEMA program exemplifies the improvement achievable through a comprehensive, evidence-based care model by integrating advanced therapies such as SGLT2 inhibitors and GLP-1 receptor agonists. It has also significantly enhanced outcomes in weight, blood pressure, glycemic control, and cholesterol levels, while increasing prescription rates for these treatments threefold [326]. The successful adoption and dissemination of a similar patient care paradigm remains a critical priority. Figure 2 summarizes how diabetic atherosclerosis can be targeted through SGLT2 inhibitors, GLP-1 receptor agonists, and nonsteroidal MRAs.

## 7. Conclusions

In conclusion, ample evidence supports that atherosclerosis in diabetes is intimately linked to complex molecular pathways involving endothelial dysfunction, oxidative stress, inflammation, and metabolic dysregulation. Therapeutic strategies targeting these core mechanisms—from SGLT2 inhibitors and GLP-1 RAs to MRAs—have demonstrated meaningful benefits in slowing atherosclerosis progression and mitigating cardiovascular risks. In DKD, these agents extend protection beyond lowering glucose by preserving endothelial integrity, modulating immune responses, enhancing lipid profiles, and improving insulin sensitivity. Preclinical and clinical findings increasingly validate their role in reducing major adverse cardiovascular events, with further promise shown by an integrated, “four-pillar” approach that combines SGLT2 inhibitors, GLP-1RAs, nsMRAs, and traditional RAAS blockade. Moving forward, more comprehensive trials are needed to refine optimal drug combinations and confirm additive or synergistic effects across different patient subgroups, particularly those with advanced diabetic kidney disease. Future research should also continue to elucidate how microbiome modulation, inflammation reduction, and improved vascular homeostasis contribute to the cardioprotective outcomes observed. These findings indicate a distinct shift in our approach to diabetes-related vascular complications, underscoring the value of multifaceted interventions that preserve renal and cardiovascular health.

## Figures and Tables

**Table 1:** Effects of GLP-1 receptor agonists on atherosclerotic plaque development.

Stage of Atherosclerosis	Effects of GLP-1 RAs	Key Mechanisms
Endothelial Dysfunction	Enhances endothelial function	↑ NO production, ↓ oxidative stress (↓ ROS), activation of antioxidant pathways (Nrf2), prevention of endothelial senescence
Inflammation	Reduces systemic and vascular inflammation	Inhibition of NF-κB, suppression of NLRP3 inflammasome, ↓ proinflammatory cytokines (TNF-α, IL-6, IL-1β), ↓ adhesion molecules (VCAM-1, ICAM-1)
Monocyte Recruitment and Macrophage Activation	Limits monocyte adhesion and promotes anti-inflammatory macrophage polarization	↓ MCP-1, VCAM-1, ICAM-1, shift from M1 (proinflammatory) to M2 (anti-inflammatory) macrophages, ↓ foam cell formation
Lipid Accumulation and Oxidation	Improves lipid metabolism and reduces ox-LDL uptake	↓ LDL-C and triglycerides, ↓ ACAT1 (cholesterol esterification), ↓ CD36 (ox-LDL receptor)
Foam Cell Formation	Prevents macrophage-derived foam cell formation	↓ ox-LDL uptake, ↓ intracellular cholesterol storage, inhibition of cAMP/PKA signaling
Smooth Muscle Cell (SMC) Proliferation	Inhibits excessive vascular smooth muscle cell (VSMC) proliferation and migration	Activation of AMPK pathway, ↓ VSMC proliferation and migration, ↓ intimal thickening
Plaque Stability	Strengthens fibrous cap and enhances plaque stability	↑ collagen deposition, ↓ MMP activity (MMP-2, MMP-3, MMP-9, MMP-13), ↓ necrotic core size
Thrombosis	Reduces platelet activation and aggregation	↑ cAMP/PKA activity, ↓ thrombus formation, enhanced antithrombotic signaling

GLP-1 RAs, glucagon-like peptide-1 receptor agonists; NO, nitric oxide; ROS, reactive oxygen species; Nrf2, nuclear factor erythroid 2-related factor 2; NF-κB, nuclear factor kappa-light-chain-enhancer of activated B cells; TNF-α, tumor necrosis factor-alpha; IL, interleukin; MCP-1, monocyte chemoattractant protein-1; VCAM-1, vascular cell adhesion molecule-1; ICAM-1, intercellular adhesion molecule-1; ox-LDL, oxidized low-density lipoprotein; LDL-C, low-density lipoprotein cholesterol; ACAT1, acyl-coenzyme A: cholesterol acyltransferase-1; AMPK, AMP-activated protein kinase; MMP, matrix metalloproteinase; VSMC, vascular smooth muscle cell; cAMP, cyclic adenosine monophosphate; PKA, protein kinase A. ↓ deacrease; ↑ increase.

**Table 2:** Key clinical trials evaluating the cardiovascular outcomes of GLP-1R agonists.

Trial Name	Drug	Population	Follow-Up Duration	MACE Outcomes
ELIXA	Lixisenatide	6068 patients with T2DM with a recent acute coronary event	2.1 years	Non-inferior to placebo (13.4% vs. 13.2% MACE; HR: 1.02; 95% CI: 0.89–1.17; *p* < 0.001 for non-inferiority, *p* = 0.81 for superiority)
LEADER	Liraglutide	9340 patients with T2DM, 81.3% with established CV disease	3.8 years	Superior to placebo (HR: 0.87; 95% CI: 0.78–0.97; *p* < 0.001 for non-inferiority, *p* = 0.01 for superiority)
SUSTAIN-6	Semaglutide	3297 patients with T2DM with established CV or chronic kidney disease	2.1 years	Superior to placebo (HR: 0.74; 95% CI: 0.58–0.95; *p* < 0.001 for non-inferiority, *p* = 0.02 for superiority)
EXSCEL	Exenatide	14,752 patients with T2DM, 73.1% with previous CV disease	3.2 years	Non-inferior to placebo for safety (*p* < 0.001); not superior for efficacy (*p* = 0.06)
HARMONY OUTCOMES	Albiglutide	4731 patients with T2DM with established CV disease	1.6 years	Superior to placebo (HR: 0.78; 95% CI: 0.68–0.90; *p* < 0.0001 for non-inferiority, *p* = 0.0006 for superiority)
REWIND	Dulaglutide	9901 patients with T2DM, 31.5% with previous CV disease	5.4 years	Superior to placebo (HR: 0.88; 95% CI: 0.79–0.99; *p* = 0.026)
PIONEER 6	Oral Semaglutide	3183 patients with T2DM, 84.7% aged ≥50 with CV or chronic kidney disease	1.3 years	Non-inferior to placebo for MACE (HR: 0.79; 95% CI: 0.57–1.11; *p* < 0.001 for non-inferiority, *p* = 0.17 for superiority)
SELECT	Semaglutide	17,604 overweight/obese individuals without diabetes and with established CV disease	3.3 years	Superior to placebo (HR: 0.80; 95% CI: 0.72–0.90; *p* < 0.001)

GLP-1RA, glucagon-like peptide-1 receptor agonist; T2DM, type 2 diabetes mellitus; CV, cardiovascular; CKD, chronic kidney disease; MACE, major adverse cardiovascular events; HR, hazard ratio; CI, confidence interval.

**Table 3:** Major adverse cardiovascular event outcomes in key SGLT2 inhibitor trials for type 2 diabetes.

Author/Year	Number of Patients	SGLT2-i	Effect on MACE
Mahaffey et al., 2018 [262]	10,142	Canagliflozin	Reduction in MACE in both primary and secondary prevention groups
Okuma et al., 2020 [263]	10,142	Canagliflozin	Significant lower risk of MACE across various BMI categories
Wiviott et al., 2019 [264]	17,160	Dapagliflozin	Insignificant reduction in MACE
Cahn et al., 2020 [265]	17,160	Dapagliflozin	Reduction in MACE was not significant
Inzucchi et al., 2020 [267]	7020	Empagliflozin	Reduction in risk of 3-point MACE

MACE (major adverse cardiovascular events) generally includes cardiovascular death, nonfatal myocardial infarction, or nonfatal stroke; however, definitions can vary slightly across trials. The CANVAS Program comprises both the CANVAS and CANVAS-R studies, while DECLARE–TIMI 58 and EMPA-REG OUTCOME are independent trials focusing on dapagliflozin and empagliflozin, respectively.
